# Going digital: added value of electronic data collection in 2018 Afghanistan Health Survey

**DOI:** 10.1186/s12982-021-00106-3

**Published:** 2021-11-24

**Authors:** Christina Mergenthaler, Rajpal Singh Yadav, Sohrab Safi, Ente Rood, Sandra Alba

**Affiliations:** 1 KIT Royal Tropical Institute, Mauritskade 64, 1092 Amsterdam, The Netherlands; 2 Particip GmbH, Merzhauser Str. 183, 79100 Freiburg, Germany

**Keywords:** Digital data collection, Tablets, Household survey, OpenDataKit, Afghanistan

## Abstract

**Background:**

Through a nationally representative household survey in Afghanistan, we conducted an operational study in two relatively secure provinces comparing effectiveness of computer-aided personal interviewing (CAPI) with paper-and-pencil interviewing (PAPI).

**Methods:**

In Panjshir and Parwan provinces, household survey data were collected using paper questionnaires in 15 clusters, and OpenDataKit (ODK) software on electronic tablets in 15 other clusters. Added value was evaluated from three perspectives: efficient implementation, data quality, and acceptability. Efficiency was measured through financial expenditures and time stamped data. Data quality was measured by examining completeness. Acceptability was studied through focus group discussions with survey staff.

**Results:**

Survey costs were 68% more expensive in CAPI clusters compared to PAPI clusters, due primarily to the upfront one-time investment for survey programming. Enumerators spent significantly less time administering surveys in CAPI cluster households (248 min survey time) compared to PAPI (289 min), for an average savings of 41 min per household (95% CI 25–55). CAPI offered a savings of 87 days for data management over PAPI.

Among 49 tracer variables (meaning responses were required from all respondents), small differences were observed between PAPI and CAPI. 2.2% of the cleaned dataset’s tracer data points were missing in CAPI surveys (1216/ 56,073 data points), compared to 3.2% in PAPI surveys (1953/ 60,675 data points). In pre-cleaned datasets, 3.9% of tracer data points were missing in CAPI surveys (2151/ 55,092 data points) compared to 3.2% in PAPI surveys (1924/ 60,113 data points).

Enumerators from Panjsher and Parwan preferred CAPI over PAPI due to time savings, user-friendliness, improved data security, and less conspicuity when traveling; however approximately half of enumerators trained from all 34 provinces reported feeling unsafe due to Taliban presence. Community and household respondent skepticism could be resolved by enumerator reassurance. Enumerators shared that in the future, they prefer collecting data using CAPI when possible.

**Conclusions:**

CAPI offers clear gains in efficiency over PAPI for data collection and management time, although costs are relatively comparable even without the programming investment. However, serious field staff concerns around Taliban threats and general insecurity mean that CAPI should only be conducted in relatively secure areas.

## Background

As of 2021, data for complex household surveys, including Demographic and Health Surveys (DHS), are often still collected using paper, which demands substantial material resource procurement, organizational planning around paper storage, and transportation and entry into the final electronic systems. This has consequences in terms of time, human and financial resources, and seriously tests the capacity of research groups implementing the survey. There is ample evidence of the benefits of computer-aided personal interviewing (CAPI) with tablets and mobile phones on data quality in high-income countries, and increasingly so in low and middle-income country (LMIC) settings as well [4–6, 10, 11]. While literature highlights CAPI’s many advantages, the upsides may not always outshine the practical hurdles, for example the time required to develop needed technological literacy; or getting past sometimes skeptical attitudes towards technology amongst survey staff, respondents, and affected communities. Researchers in fragile and unstable settings who are strapped for time and resources do however recognize that CAPI can seriously reduce this strain, yet there is somewhat limited data comparing the performance of PAPI versus CAPI in fragile and conflict-affected settings [8]. From the standpoint of public health research, and to understand factors affecting the uptake of CAPI in insecure settings, we evaluated the differences in time and cost efficiency of training, procurement and data collection; data quality; and user perceptions of these two methods. The 2018 Afghanistan Health Survey, which aimed to describe key maternal, child and general population health metrics and access issues, provided an opportunity to compare factors affecting uptake of CAPI versus those for which PAPI is better suited. The full report of the survey is available online (‘[3]’, no date), while the aim of the study presented in this manuscript was to assess potential added value of CAPI over PAPI from three perspectives: efficiency of implementation, data quality, and user and respondent acceptability.

**Fig. 1:**
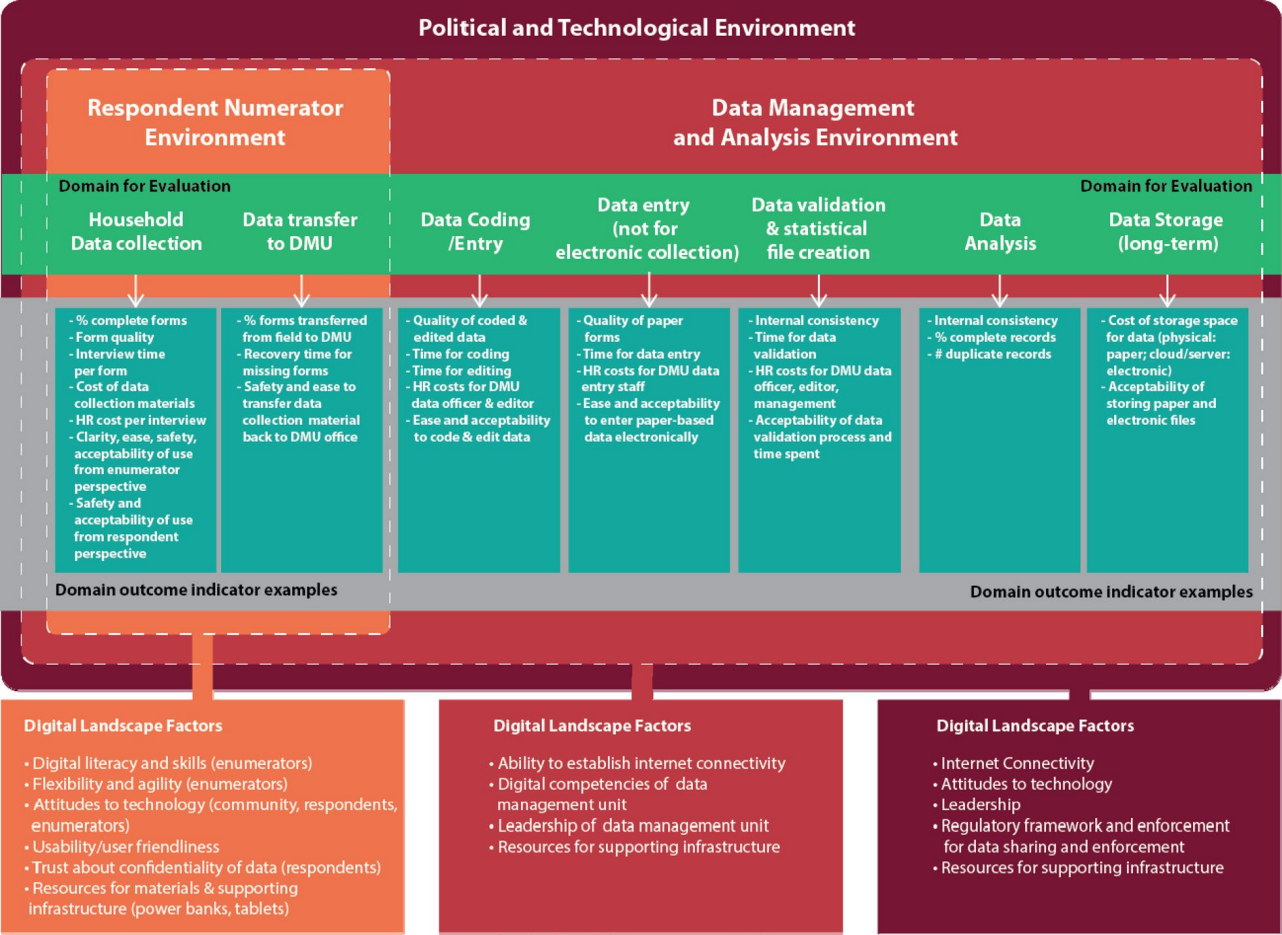
Conceptual framework to evaluate digital technology uptake in survey settings

Investigators of the Digital Engagement and Resilience (DEAR) project of University of Aberdeen described the influence of societal and information technology ecosystem factors on the uptake of digital technology at the individual and group level; internet infrastructure, access to digital technology, digital literacy and attitudes each affect the likelihood that CAPI can benefit users and data quality to the extent documented in ideal conditions [13]. Danksy et al. recognized the same and developed a framework to evaluate how various environmental factors challenge or enable use of the technology and acceptability of users [7]. Based partially on a number of factors from the DEAR project framework, Smith et al.’s Total Survey Error (TSE) framework [15] and the existing body of research we developed a theoretical framework to evaluate the capacity to uptake digital technology in a survey setting, across three concentric environments (Fig. 1). This framework intends to capture the factors which affect the quality, efficiency and acceptability of digital data uptake from the stage of household data survey collection to data analysis and broad-scale dissemination, and is especially useful in fragile or insecure settings. While a number of online resources provide guidance on the use of tablets to collect and analyse data [14, 17], limited studies document issues around acceptability and feasibility in a rigorous way [10].

The 2018 Afghanistan Health Survey was conducted in all 34 provinces of the country to collect nationally representative data on household sociodemographic, maternal and child health indicators. To the best of our knowledge, this is the first nationally representative survey with digital components in Afghanistan. Data collection was done using paper forms throughout the country except for two relatively secure provinces Panjshir and Parwan, where study coordinators aimed to measure the added value of CAPI compared to PAPI by training enumerators to administer the survey using tablet computers. More secure settings were chosen for this study as the Taliban has been known to capture and interrogate field researchers and health workers at security checkpoints [2], particularly those with valuable equipment such as laptops or tablets [16].

Our objective was to evaluate data efficiencies, user acceptability and feasibility in the 2018 Afghanistan Health Survey across the three environments of digital technology uptake: the respondent—enumerator environment, the data management and analysis environment, and the political and technological environment. The aim of this study is provide evidence for the scale up of digital survey technology in future surveys in Afghanistan and other fragile and conflict affected settings.

## Methods

**Fig. 2:**
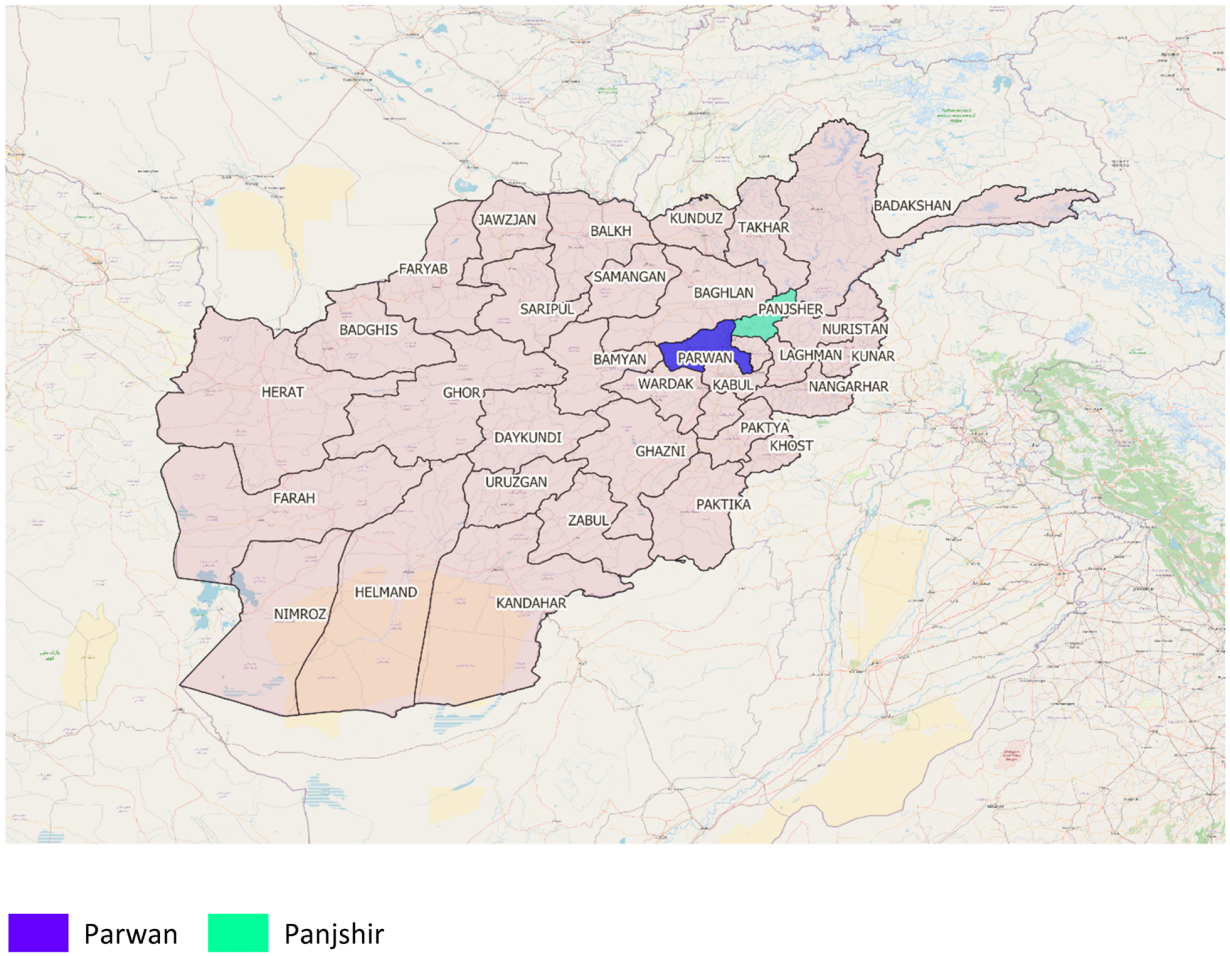
Location of Panjsher and Parwan ODK study sites in 2018 Afghanistan Household Survey

For each stage of the framework we developed we determined which indicators should be evaluated to measure this uptake. For the Afghanistan Health Survey we applied a two-stage cluster design with the first stage as the province consisting of 15 clusters per province, and the second stage as cluster with 23 households per cluster. To compare efficiency, data quality and acceptability of PAPI versus CAPI, we carried out a mixed methods study in two of the 34 provinces: Panjshir and Parwan (Fig. 2). We used both quantitative reanalysis of survey processes and output data as well as qualitative analysis of survey staff focus group discussions.

In both Panjshir and Parwan, household survey data were collected on paper questionnaires in 15 clusters, and using electronic data collection software (in our case OpenDataKit [ODK]) installed on electronic tablets in the remaining 15 clusters. The data management unit (DMU) was responsible for electronic entry of paper-based data, data coding, verification and validation. The DMU was led by a data manager and deputy data manager, data entry supervisor, data officer, 20 data entry operators, and five data coding officers.

### Timeliness and cost efficiency

We assessed time taken and expenditures made to collect and process data to determine whether any efficiencies were gained by collecting data electronically. For this purpose we reviewed detailed financial expenditure reports involved in preparation and execution of the household survey in Panjshir and Parwan provinces. We analysed time stamps for key data collection and data management steps: (1) start and stop time stamps for each of six household survey modules and; (2) start and stop time stamps for data coding, first data entry and second (verification) data entry. Data collection time stamps were available in the study databases as these were automatically collected in the data collection software or manually written in the paper-based household survey questionnaires. Data coding and entry time stamps were manually logged by data management staff for paper-based questionnaires only. We used these to calculate hours spent coding and entering data and subtracted one hour from each day worked to account for breaks and rest. CAPI did not require substantial time investments for data coding, entry or verification because they were already collected and stored electronically on the study’s server database (ODK Aggregate in our case), so no direct comparison between the time spent coding and verifying paper and digitally collected data could be made. Calculations around human resource time factored in hours worked by the entire DMU team.

### Data completeness and consistency

Data quality was measured by examining completeness of data points (i.e. the number of household respondents multiplied by the number of expected responses per respondent) in the pre-cleaned database and the final database of both the PAPI and CAPI. The pre-cleaned database contains the doubly entered electronic data from the paper-based surveys, and the raw data uploaded to the server from electronic tablets. The final database contains the data which, when collected on paper, were then coded and doubly entered, and when electronically collected, were also coded and then received further cleaning (i.e. merging household members and de-duplicating). Due to survey skip logic, household respondents were asked different numbers and combinations of questions. We standardized the measure of completeness across all respondents by studying the questions which everybody was asked. Therefore we identified forty-nine tracer questions which the survey required from each respondent out of a total of 295 possible survey questions, regardless of any answers they supplied for any other questions. Per household, we calculated the ratio of complete tracer data points to total possible tracer data points in the pre-cleaned and final paper and electronic datasets, for each survey module. Data cleaning removed some data points from the final datasets of both ODK and paper-based data, for example those data which were collected due to incorrectly applied skip logic, or were clearly illogical.

### Acceptability

We compared acceptability of paper-based and electronically collected data from the perspective of survey field staff; in total there was one team per province consisting of three male and three female enumerators, one supervisor, and for paper clusters only one data editor. We conducted three focus group discussions (FGD): one female and one male group, each with four enumerators and one field editor, and one separate FGD consisting of four field team supervisors (two female and two male). In each FGD, the survey staff present conducted both the paper-based surveys and electronic survey clusters. Focus groups were interviewed in a semi-structured fashion about perceptions of user-friendliness, difficulties in the field, efficiency, skip logic, safety of use, and enumerator-respondent interactions. We later administered a two-question survey about safety and comfort during digital data collection to field staff trainees for two subsequent nationally representative surveys (HMIS verification and BSC), to get the perspectives for insecure areas. As the focus group discussions occurred after the household survey and operational research and all payments had been made to study staff, we had negligible concern that respondents felt compelled to respond in a specific way. Therefore possible effects of the interviewer on respondents were not accounted for.

## Results

### Are electronic household clusters’ data collected more efficiently?

#### Timeliness

**Fig. 3:**
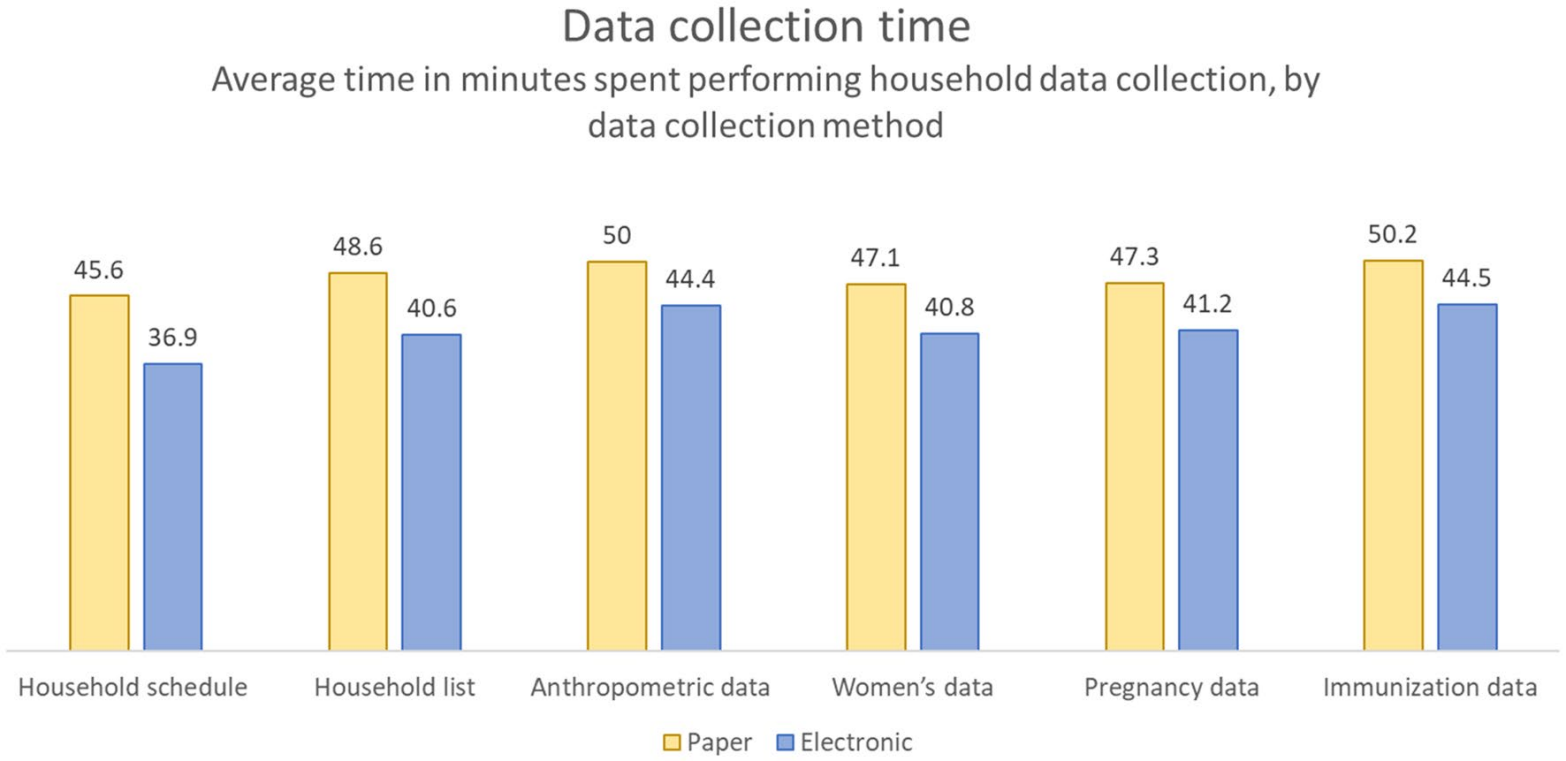
Average time in minutes spent performing household data collection: Paper vs. Electronic

For each individual module, enumerators in electronic cluster households spent significantly less time administering the questionnaire, for an average time savings of 7.4 min per module per household (95% CI 6.6–8.2 min) (Fig. 3). Comparing the time required to administer the entire survey in a household, enumerators also spent significantly less time administering them in electronic cluster households (248 min total survey investment time [95% CI 239.1–258.1]) compared to paper clusters (289 min [95% CI 277.8–299.8]), for an average savings of 40 min per household (95% CI 25.4–55.1 min).

Paper-based questionnaires contained responses which needed to be coded into categories to allow for data analysis. Parwan DMU staff spent an average of 5 min and 54 s per household to code responses, while in Panjshir DMU staff spent an average of 8 min and 33 s to code responses per household. The first entry from paper into the electronic database of one Parwan household of data required an average of 18 min and 40 s; the verification entry required an average of 14 min and 55 s. Panjshir’s first entry household average time was 13 min and 40 s, while the verification data entry required an average of 13 min and 16 s. Additional quality control performed by two lead DMU staff required approximately 9 eight-hour working days, or an average of 18 min per household.

**Fig. 4:**
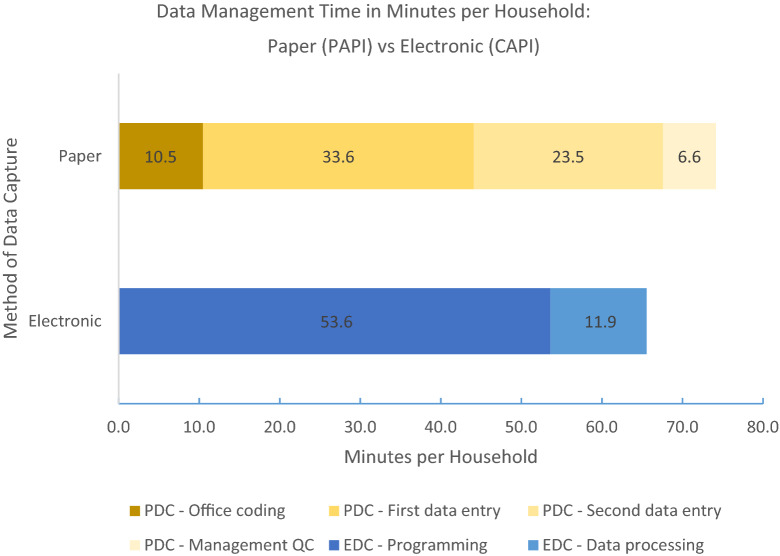
Time performing data management tasks per household: Paper vs. Electronic

Programming electronic surveys for use in ODK software lasted approximately 73.5 eight-hour working days. Programmers estimated that 30% of these hours were spent revising skip logic and survey screen formatting to accommodate different questionnaire requirements for male and female respondents. 10% of these hours were spent translating the survey into Dari language. Data were transferred to the server database using WiFi hotspots installed via tablet sim cards. Coding the electronically collected data required two eight-hour working days, and no verification entry was performed or necessary. Additional data cleaning and quality control performed by two lead DMU staff required about 7 eight-hour working days each. Figure 4 presents the time used for all data management tasks (programming and quality control for ODK; coding, data entry and quality control for paper) on a household level. Average time spent for all data management tasks was approximately 74 min for paper-based households and 66 min for ODK households. Total data management for ODK data took a total of 719 h (90 person-days), only 131 h (16.4 person-days) of which were spent for processing (coding and cleaning). PAPI data coding, entry, management and cleaning alone consumed a total of 812 h (101.5 person-days).

#### Cost efficiency

The actual combined expenditures in personnel fees and transportation, data management, training, procurement of equipment, and printing amounted to a per province total of 59,958 USD spent for the combined electronic clusters, and 35,762 USD spent for the combined paper clusters (Table 1). Field team personnel costs (fees and transportation) comprised the majority of these costs at 26,100 USD in both paper and electronic clusters, covering six interviewers, one data editor, and one supervisor for each province. These teams of eight conducted surveys in both the paper and electronic clusters of each province. The largest cost difference resulted from the investment required to electronically program the survey, increasing the cost for CAPI clusters by 19,634 USD. Costs to develop the survey questionnaires into a word processor were not factored into this analysis. Training costs for CAPI staff exceeded those of the PAPI staff by 2826 USD because they received both PAPI and CAPI training. This was necessary because CAPI staff also performed paper-based surveys in the remaining 32 provinces of Afghanistan, and these skills certainly contributed to the quality and efficiency of the work performed by survey staff in the CAPI clusters. Equipment costs of 2157 USD for CAPI cluster tablets and charging devices were not required in the paper clusters, which only had an additional 516 USD of printing costs for the paper forms not needed in the electronic clusters. Per household the CAPI method cost 91.12 USD, while PAPI methods cost 54.43 USD per household.

**Table 1:** Cost comparison of PAPI and CAPI data collection and management

Line item	PAPI clusters	CAPI clusters	CAPI clusters without upfront programming costs
Field team personnel (fees and transportation) costs			
Interviewers (six people per province)	$13,500.00	$13,500.00	$13,500.00
Editor (one person per province)	$2,700.00	$2,700.00	$2,700.00
Supervisor (one person per province)	$9,900.00	$9,900.00	$9,900.00
Total	$26,100.00	$26,100.00	$26,100.00
Data management costs			
Survey programming costs	$0.00	$19,634.40	$0.00
Data entry and coding costs	$1,082.25	$0.00	$0.00
Data cleaning and management costs	$1,256.40	$2,432.65	$2,432.65
Total	$2,338.65	$22,067.05	$2,432.65
Training costs			
Standard training cost to AHS field staff	$6,807.33	$6,807.33	$6,807.33
Electronic data collection training for Panjshir and Parwan	$0.00	$1,413.19	$1,413.19
Electronic data collection refresher training for Panjshir and Parwan	$0.00	$1,413.19	$1,413.19
Total	$6,807.33	$9,633.71	$9,633.71
Equipment costs			
Costs of tablets for electronic data collection	$0.00	$1,729.41	$144.12
Costs of power banks	$0.00	$247.06	$20.59
Costs of internet for data transfer	$0.00	$40.00	$3.33
Costs of umbrellas	$0.00	$17.65	$1.47
Costs of other equipment	$0.00	$123.53	$10.29
Total	$0.00	$2,157.65	$179.80
Printing costs			
Printing costs for paper survey forms	$516.18	$0.00	$0.00
Costs of photocopying paper	$0.00	$0.00	$0.00
Total	$516.18	$0.00	$0.00
Data collection type total	$35,762.16	$59,958.41	$38,346.17
Data collection type total per household:	$54.43	$91.12	$58.28

When initial costs associated with CAPI are removed (one-time survey programming and equipment costs), the total cost for CAPI would have been 38,346 USD. This however does not account for efficiencies which would be gained over time for data cleaning and management, and includes one replacement tablet, power bank, an internet package, umbrella and other equipment. The per household costs would also be more comparable to the PAPI costs at 58.28 USD.

### Are electronically collected data of higher quality than data collected on paper?

#### Data completeness

**Fig. 5:**
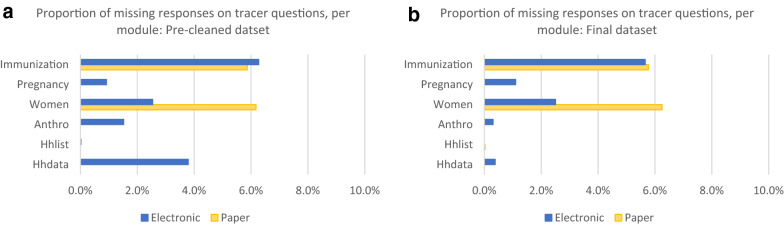
**a** Pre-cleaned dataset completeness for Paper and Electronic. **b** Final dataset completeness for Paper and Electronic

Differences in data quality were evaluated from the perspective of data completeness. In each module we calculated the number of missing data points out of all possible required data points for the forty-nine tracer variables only. For the pre-cleaned data (as defined above), the paper-based dataset was missing a slightly higher percentage of tracer data points (2.0%: 1202/59,424) compared to the electronically collected dataset (1.6%: 911/55,576) (Fig. 5a).

After data cleaning, the electronically collected dataset was still missing slightly fewer tracer variable data points (1.1%: 626/55,466) than the paper dataset (2.0%: 1223/59,980) (Fig. 5b). In both the pre-cleaned and final datasets paper-based data only modules on women’s health and immunization were lacking any tracer data. The electronically collected dataset missed tracer data points in all modules, but primarily in the women’s and immunization modules. One question related to children’s vaccination cards was unexpectedly routinely skipped, accounting for more than 95% of the missing data points in the immunization module.

### How acceptable did field enumerators and respondents find electronic data collection compared to paper based data collection?

#### Clarity and ease of use

Data collectors were interviewed about clarity and ease of using electronic versus paper forms for data collection. Most enumerators preferred electronic data collection over paper due to substantial time savings, user-friendliness, improved data security, and less conspicuity when traveling. These benefits of CAPI were cited in all three focus group discussions. Automated skip logic was the most frequently mentioned reason for preferring CAPI. This removed the necessity to manually decide which questions need to be asked based on previous responses, as the electronic program did this for data collectors. Many enumerators mentioned that this was a helpful time-saver during data collection.

Enumerators also appreciated the logic constraints of CAPI, and that certain fields did not make it possible to enter impossible answers, for example an age which is too high or too low. In terms of practical convenience, some enumerators reported that tablets were preferable to paper when it was raining, as papers would sometimes become wet and difficult to read, while a tablet is easier to protect from rain. Data collectors also enjoyed that tablets were lighter to carry than large piles of paper questionnaires.

Some limitations around CAPI were noted. A few data collectors reported that previously entered information appeared to sometimes be erased when they scrolled back in the questionnaire to correct responses. One enumerator noted that when the tablet memory was full, tablets would turn off, and sometimes tablets would just turn off for no apparent reason. Several enumerators mentioned that when they only recognized mistakes after multiple incorrect or irrelevant responses were entered into the tablet, it was burdensome to remove all incorrect data from the electronic form. With paper it is easy to simply disregard some incorrect responses by crossing them out and writing the correct answer on the spot. CAPI moves up this type of data cleaning to the field rather than the data management office.

#### Interactions with household respondents

Many of the enumerators mentioned that household respondents were skeptical about safety of their data when they would be entered into tablets. Some household respondents were concerned that the tablets may be used to photograph them or record their voices. This was more of a concern among women than among men. However, they also mostly reported that these concerns were easily resolved by enumerator reassurance about electronic data confidentiality. Conversely some household respondents (generally younger) felt more secure about their information being entered into a tablet rather than paper, because they understood that it would be more difficult for people to get a password to open up the questionnaires on tablets, than it would be to read responses on paper. 54% (80/148) of enumerators in a follow-up survey reported that they would not feel safe and comfortable if they had to use tablets for household data collection. Among those who reported not feeling safe, 22% (n = 18) mentioned Taliban concerns, 33% (n = 26) mentioned concerns around insecurity, 19% (n = 15) mentioned that as tablets are known to collect GPS coordinates, this makes them feel vulnerable to retaliation from either Taliban or communities, and 33% (n = 26) reported that household and community members would be hesitant to accept this practice. Among those working in provinces in which there was Taliban occupancy in 2019 (n = 88), 47 (53%) anticipated not feeling safe collecting data in households in the future.

#### Safety concerns

When asked which method of data collection they would prefer to use for a hypothetical next survey, most enumerators indicated that CAPI is preferred when possible. It was perceived as much more comfortable than PAPI, more convenient, and by some respondents it was perceived to be more safe even in insecure areas. One can more easily conceal tablets whereas it is often more difficult to hide a large stack of paper questionnaires. The focus group discussions revealed that some enumerators felt unsafe carrying tablets though due to fears that people may steal them. The two-question survey administered to field staff trainees in all 34 provinces provided more insight into security concerns: 48% (72/148) of enumerators reported that they did not feel safe and comfortable when they used tablets for data collection in a recent health facility survey. Among the 72 respondents who reported not feeling safe, 79% (n = 57) say they are concerned about repercussions from Taliban who view use of smart devices with suspicion due to their capacity to capture GPS coordinates, and subsequent future retaliation from either Taliban or communities. 46% (n = 33) mentioned concerns around general insecurity or theft. Among those working in provinces with Taliban occupancy (n = 88), 38 (43%) reported feeling unsafe using tablets to collect data in health facilities. Enumerators who reported feeling unsafe using tablets in a health facility were 4.5 times more likely (95% CI 2.9–7.2) to report anticipate feeling unsafe doing so in a household setting. A few enumerators also mentioned that collecting data electronically in the future depends on the skills of the survey staff. One enumerator mentioned that the only advantage of using paper in the future is that communities more quickly/ easily recognize and accept PAPI.

## Discussion

This study offers a systematic assessment of widely accepted indicators of data quality and efficiency within the frame of digital engagement in a conflict-affected setting. Well-maintained original and final databases, and rigorous record-keeping around costs incurred and hours logged were key to the success in comparing quality, efficiency and feasibility of CAPI versus PAPI in this survey setting. This study suggests that even in fragile and conflict-affected settings, data collected through CAPI can show improved quality and efficiency of collection and management compared to PAPI, although initial programming investments CAPI are costly. Even without upfront programming costs, CAPI was 2584 USD more expensive than PAPI, although these could likely be mitigated due to efficiencies gained from increased experience with the data collection software and by consolidating trainings. Caeyers described a number of detailed scenarios which make CAPI more cost-effective than PAPI elsewhere [6]. For repeat surveys in secure settings, electronic programming is preferable as it brings the data management unit more efficiently to the desired end state of electronic storage, even if it is slightly more expensive.

In our study an average time savings of 40 min per household was observed in CAPI clusters compared to paper, and when comparing data management and cleaning time alone, 87 person-days overall (14.5 compared to 101.5) were saved in CAPI clusters. A comparison of CAPI and PAPI in households in Burkina Faso showed similar results for saved time in households [9]. Both pre-cleaned and final electronic collected datasets were slightly (< 1%) more complete than their paper counterparts, which is comparable to findings from similar comparisons in a Kenyan study with 1% and 0.1% missing data in paper and electronic datasets respectively [12]. The Burkina Faso study found negligible differences in completeness but duplicates only in paper datasets [9]. A comparison of PAPI with tablet-based CAPI in northwest Ethiopia also found data completeness to be superior in the electronically collected data [19]. The same study showed that enumerators favored CAPI to PAPI for similar reasons to those of our study: improved efficiency of data collection, improved quality due to automatic skip patterns and data quality checks, faster data transfer and tablets are more convenient to transport.

However, CAPI costs exceeded PAPI by 68%, but this was almost entirely due to the upfront electronic programming costs of 19,634 USD. These CAPI costs far exceeded what other studies have shown. A Kenyan study assessing costs of PAPI and CAPI found that establishment of a smartphone data collection system was 9.4% more expensive than PAPI, yet over the subsequent two years, CAPI was 7% less expensive to operate compared to PAPI [12]. The same Kenyan study also reported that enumerators appreciated the opportunity to improve their IT skills through CAPI [19]. Despite the similarity in costs, in the long run, investment in CAPI may be a worthwhile investment in relatively secure areas to save data collection time in the field, and data management time in the office. Over time, trainings may become shorter as trainees become increasingly familiar with digital technology, and for repeat surveys, programming time should remain very low. Time is also a valuable resource.

Interviews on acceptability highlighted a number of relevant concerns around the use of digital data in fragile settings: threats to the person(s) carrying the tablet and respondent hesitations were discussed in equal measure. Similar concerns were raised by enumerators of the Ethiopian study [19], and by researchers in a South African study [18]. These issues raise the need to mitigate security risks for field staff using electronic devices in fragile settings and to pre-sensitize communities about safe storage of electronic data in these settings. Local leaders are often engaged prior to household surveys for a similar purpose, yet sharing in advance the news that field staff will pass through an area with valuable electronic devices may further expose them to risk [18].

The efficiencies and user acceptance described above add evidence to the argument for further expansion of digital technology in survey settings, also those affected by insecurity. But total replacement of digital technology over paper is unlikely to occur quickly according to Adner and Kapoor’s ‘War Between Ecosystems’ paradigm, in which the two systems enjoy a ‘robust coexistence’ dynamic [1]. This particular ecosystem enables both the piloting of digital systems where it is safe to do so, and the use of the ‘old technology’ of paper. Survey planners are well acquainted with the process of developing paper-based questionnaires and electronically entering paper data, which have been extensively documented in detailed methodological open-source tools and guidelines. Current trends in the technological and political environment suggest that Afghanistan will remain in quadrant two, meaning that digital technology is unlikely to replace paper in the coming years. Challenges abound: digital data collection issues like accessing stable internet connectivity, programming forms, and resolving technological errors usually require specialized knowledge and training. In addition, insecurity also undoubtedly slows down progress towards the next ecosystem evolution.

### Limitations

A number of study limitations affected the results presented here. Because the study was conducted as an operational research add-on to an existing household survey, we did not prioritize randomization of household clusters to the CAPI and PAPI groups. Therefore we cannot attribute differences to the mode of collection. However the qualitative data does provide some evidence that improvements in timeliness can be attributed to CAPI, as data collectors found CAPI to reduce time spent per household. A systemic error affected the calculation of a question in the immunization module. An incorrect skip pattern in the paper questionnaire and subsequent incorrectly programmed skip logic in the electronic version meant that about 40% of responses were missed for this question. A similar mistake led to high missingness for a family planning question in the women’s module. Additionally, time stamps for coding and data entry for paper data were not logged using a precise and consistent approach across all DMU staff, so logged hours could have included non-coding or data entry tasks.

CAPI and PAPI cluster costs are also difficult to compare because of several factors. Because survey programming was carried out by developers with highly specialized skills, programming costs calculated for CAPI clusters far outweighed the overall data management costs of PAPI clusters. Also, an estimated 30% of CAPI programming time was spent on revisions, adding substantial costs which theoretically could have been avoided if all formatting specifications were clear upfront. But in practice achieving perfect tool design at the first opportunity is often unrealistic due to challenges which arise during tool design. Finally, due to the high upfront costs to program CAPI questionnaires, costs for CAPI data management and in total appear much higher than PAPI, although CAPI still offered considerable time savings. Caeyers et al. suggested a simple formula to calculate the threshold number of forms above which CAPI becomes cost-effective enough to justify programming expenses, and pointed out that if a repeated or modified version of the survey is anticipated, this should prompt planners to consider CAPI [6].

Furthermore, future studies would benefit from including four additional indicators: internal consistency of the final dataset, number of duplicate records, timeliness of data availability, and time for data coding and second-step (verification) entry of electronically collected data (Table 2). Internal consistency was not monitored using a systematic approach; however, we programmed skip logic and field data type constraints into the electronic questionnaire to eliminate user error. The comparison of data completeness and anecdotal evidence suggest that CAPI performed better than paper in this regard. For example, six households in paper clusters which did not report having any children under the age of five also contained data on under five illness, while none of the electronic cluster households showed such an inconsistency. 11 respondents from paper clusters responded that a child was born dead, but later responded that the same child is still alive, while no similar irregularities were observed in the electronic cluster data. The same pattern was found in the Ethiopian study, in which invalid entries or errors were more found in nearly half of the paper-based surveys compared to one-third of the electronically collected data [19].

The level of effort required to prepare CAPI for coding and verification deserves to be underscored. CAPI still required a substantial time investment so that survey modules could be merged for analysis. Due to user error during household data collection, incorrect line numbers were sometimes entered into some modules of the household interviews, resulting in errors during merging, and software-generated record duplication. Although these issues could eventually be resolved in two forty-hour work weeks, it was occasionally necessary to seek information from the field regarding specific households and individuals to merge survey modules correctly. Even in CAPI, human error in data entry will lead to logical impossibilities during merging, which different software packages will handle differently [10]. Understanding how one’s preferred software will handle many-to-one or one-to-many merging errors is essential before using it to start managing survey data.

**Table 2:**
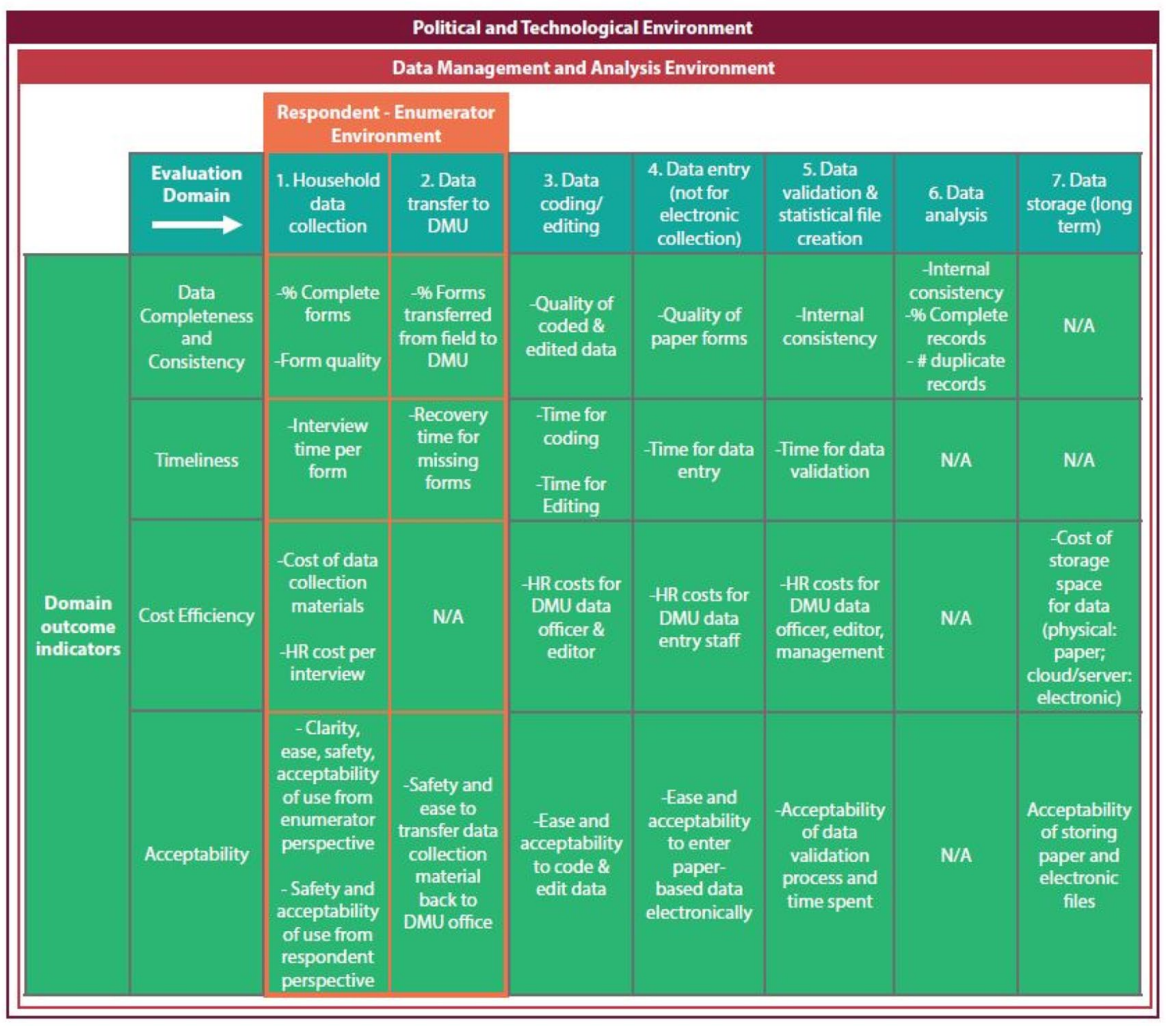
Operationalization table for evaluation of digital data collection, management and analysis

## Conclusions

The added value of CAPI over PAPI was primarily in time efficiencies gained during data collection and processing, although only conducted in relatively secure areas. CAPI also resulted in improved acceptability while preserving data quality, despite higher costs. Overall our data provide evidence to support the scale up of digital survey technology in future surveys in Afghanistan and other fragile and conflict affected settings. Hybrid approaches such as ours (in which both paper and digital data collection are deployed) may be appropriate in settings where security varies across space and time.

## Data Availability

The datasets generated and/or analysed during the current study are available in the https://zenodo.org repository, [https://zenodo.org/record/4279123#.X7UBc8hKiUk]. The Afghanistan Health Survey full report can be found available at https://www.kit.nl/wp-content/uploads/2019/07/AHS-2018-report-FINAL-15-4-2019.pdf.

## Abbreviations

CAPI: Electronic data collection
PAPI: Paper data collection
ODK: OpenDataKit
DHS: Demographic and Health Survey
AHS: Afghanistan Health Survey
DMU: Data management unit
FGD: Focus group discussion
HMIS: Health management information system
BSC: Balanced Score Card
CI: Confidence interval
USD: United States Dollars
GPS: Global Positioning System
